# Expression of Angiopoietin-2 and Vascular Endothelial Growth Factor Receptor-3 Correlates with Lymphangiogenesis and Angiogenesis and Affects Survival of Oral Squamous Cell Carcinoma

**DOI:** 10.1371/journal.pone.0075388

**Published:** 2013-09-11

**Authors:** Chao Li, Jinchuan Fan, Xicheng Song, Bing Zhang, Yu Chen, Chunhua Li, Kun Mi, Hong Ma, Yufeng Song, Xiaofeng Tao, Guojun Li

**Affiliations:** 1 Department of Head and Neck Surgery, Sichuan Cancer Hospital, Chengdu, China; 2 State Key Laboratory of Oral Diseases in Sichuan University, Sichuan, China; 3 Department of Otorhinolaryngology Head and Neck Surgery, Yuhuangding Hospital, Qingdao University, Yantai, China; 4 Department of Oral and Maxillary Surgery, the Affiliated Hospital of Guiyang Medical College, Guiyang, China; 5 Radiology Department of Shanghai Ninth People's Hospital Affiliated Shanghai JIaoTong University School of Medicine, Shanghai, China; 6 Department of Head and Neck Surgery, U.T. M.D. Anderson Cancer Center, Houston, Texas, United States of America; The University of Kansas Medical center, United States of America

## Abstract

**Background:**

Both Ang-2 and VEGFR-3 are major regulators of angiogenesis and lymphangiogenesis, respectively, and thus may affect prognosis of OSCC. We sought to determine the associations between Ang-2 and VEGFR-3 expression and survival of OSCC.

**Methods:**

Ang-2 and VEGFR-3 expression was determined immunohistochemically in tumor tissues from 112 patients with OSCC; OSCC-adjacent noncancerous oral tissue from 85 OSCC patients; and normal oral mucosa from 37 cancer-free individuals. A log-rank test and Cox proportional hazard models were used to compare survival among different groups with expression of Ang-2 and VEGFR-3.

**Results:**

Ang-2 and VEGFR-3 expression was upregulated in OSCC compared to nontumor tissue (all *P*<0.05). High Ang-2 expression positively correlated with microvessel density (MVD) (*P*<0.01), and high VEGFR-3 expression positively correlated with lymph node metastasis (*P*<0.01) and lymphatic vessel density (LVD) (*P*<0.01). The patients with high expression of Ang-2 alone or in combination with VEGFR-3 had a significantly worse survival than in patients with low expression of Ang-2 or any other co-expression status (all *P*<0.05), respectively. Furthermore, multivariable analysis showed that patients with high expression of Ang-2 alone or in combination with VEGFR-3 had a significantly increased risk of death compared with those with low expression of Ang-2 or any other co-expression status (HR, 2.7, 95% CI, 1.1–6.2 and 5.0, 1.3–15.4, respectively).

**Conclusions:**

These results suggest that increased expression in tumors of Ang-2 may individually, or in combination with VEGFR-3, predict poor prognosis of OSCC.

## Introduction

OSCC metastasizes primarily via the lymphatic system. In patients with OSCC, the presence of lymph node metastases is widely accepted as a major prognostic factor and is associated with higher recurrence rates and an approximately 50% reduction in overall survival [1].However, the mechanisms by which OSCC metastasizes to lymph nodes have been studied only very recently. Previous study reported that overexpression of angiopoietin-2 (Ang-2), which has been implicated in lymphatic vessel development [2], was closely associated with angiogenesis and lymph node metastasis in OSCC [3]. Vascular endothelial growth factor receptor-3 (VEGFR-3) is one of the first lymphatic endothelial-cell-specific cell surface molecules involved in regulation of lymphangiogenesis, and might have crucial roles in amplification of pathological lymphangiogenesis and angiogenesis [4]. However, whether altered expression of Ang-2 and VEGFR-3 affects lymphangiogenesis and survival of OSCC is not known.

To better understand the functional contribution of Ang-2 and VEGFR-3 to lymphangiogenesis and progress of OSCC, we used a double-labeling immunohistochemical staining of CD-34/D2-40 in blood vessels and lymphatic vessels of tumor specimens for determination of microvessel density and lymphatic vessel density among 112 cases. Moreover, in these tumor specimens, we also performed immunohistochemical staining of VEGFR-3, a major regulator of lymphangiogenesis [4], to investigate the correlations between Ang-2 and VEGFR-3 expression and tumor lymphangiogenesis and progress and thereby reveal the role of Ang-2 and VEGFR-3 in lymphatic metastasis and clinical survival in OSCC patients.

## Materials and Methods

### Ethics Statement

The protocol for this study was approved by the Ethics Committee of the Sichuan Cancer Hospital, Chengdu, Sichuan, China and Affiliated Hospital of Guiyang Medical College, Guiyang, Guizhou, China, and written informed consent was obtained from all study patients.

### Patients

This study included a total of 112 patients with OSCC who were treated at the above two institutions from 1998 to 2007. The patients with histopathologically confirmed OSCC, who underwent surgical resection procedures at the above mentioned two institutions, were enrolled in the present study if the patients: 1) had pathological diagnosis of OSCC by a senior pathologist and no other primary cancers; 2) were dignoised with OSCC without previous preoperative radiotherapy, chemotherapy, or hormone therapy; and 3) signed informed consent for the study. The median follow-up period for these 112 patients was 58 months (range, 3–60 months). In addition, noncancerous oral tissues 2 to 3 cm from the primary tumor were obtained from 85 patients with OSCC. Furthermore, normal oral mucosa samples were collected from 37 patients with a trauma or tooth problem and had never smoked or consumed alcohol who visited Affiliated Hospital of Guiyang Medical College from 1998 to 2005. The specimens were fixed in 100 ml/L formalin, embedded in paraffin, and then examined histologically. The study patients were categorized into different subgroups according to the 7th edition of the AJCC cancer staging manual [5] and differentiation classifications [6].

### Conventional Immunohistochemical Analysis of Ang-2 and VEGFR-3 Expression

**Figure 1:**
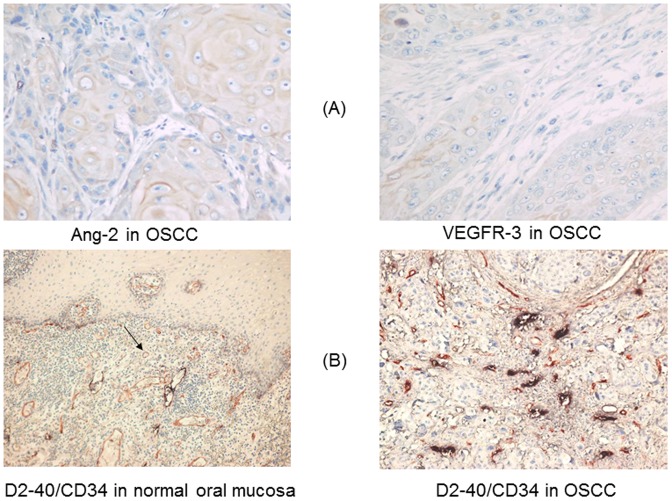
Immunohistochemical expression of Ang-2 and VEGFR-3 in OSCC (streptavidin/avidin-biotin complex ×400) (A) and CD34 (scarlet; blood vessels) and D2-40 (purple-black; lymphatic vessels) in OSCC and normal oral mucosa (streptavidin/avidin-biotin comple double-labeling ×400) (B).

Ang-2 and VEGFR-3 expression in OSCC and control tissues was evaluated using the streptavidin/avidin-biotin complex (SABC) immunohistochemical kit (Boster Biological Technology Ltd, Wuhan, China) (**Figure 1 A**). Briefly, after deparaffinization and rehydration, the histologic sections were peroxidase-blocked with 3% hydrogen peroxide for 10 min, heated in a microwave oven to retrieve the antigen twice for 10 min each, and incubated with normal goat serum for 20 min. Sections were then incubated with anti-Ang-2 monoclonal antibody (1∶80 dilution; Boster Biological Technology Ltd) or anti-VEGFR-3 monoclonal antibody (1∶90 dilution; Boster Biological Technology Ltd) overnight at 4°C. Afterward, the sections were washed with phosphate-buffered saline solution (pH = 7.4) and incubated with biotinylated secondary antibody, SABC, and 3′-3′-diaminobenzidine chromogen (Boster Biological Technology Ltd) to visualize binding of Ang-2 or VEGFR-3 antibody. Then sections were counterstained in hematoxylin, dehydrated through graded alcohols, and mounted on slides. Stained slides were then examined microscopically and scored for intensity or percentage of positive cells.

### Double-Labeling Immunohistochemical Analysis of Angiogenesis and Lymphangiogenesis

To quantitatively assess angiogenesis and lymphangiogenesis in OSCC specimens, a double-labeling immunohistochemical technique was used to simultaneously stain for CD34, a marker of vascular endothelial cells, and D2-40, a marker of lymphatic endothelial cells. D2-40 staining was performed first, followed by CD34 staining (**Figure 1 B**). Sections were washed with Tris-buffered saline solution (pH = 7.0). The deparaffinized and rehydrated tissue sections were peroxidase-blocked with 3% hydrogen peroxide for 15 min, heated once in a microwave oven for 10 min to retrieve the antigen, incubated with normal blocking serum, and then incubated with an anti-D2-40 monoclonal antibody (1∶100 dilution, ZhongSan Biological Technology Ltd) overnight at 4°C. A biotinylated secondary antibody, SABC, and its substrate 5-bromo-4-chloro-3-indolyl phosphate/nitro blue tetrazolium were used to visualize binding of the D2-40 antibody. The positive areas were purple-black.

Subsequently, endothelial cells were stained by overnight incubation at 4°C with an antibody to CD34 (1∶50 dilution, ZhongSan Biological Technology Ltd). A biotinylated secondary antibody, SABC, and its substrate 3-amino-9-ethyl carbasole were used to visualize binding of the CD34 antibody. The positive areas were scarlet.

Following CD34 and D2-40 staining, both blood vessels (CD34-positive, scarlet) and lymphatic vessels (D2-40-positive, purple-black) were visible in each histologic section.

### Quantitative Assessment of Immunostaining Intensity, Microvessel Density, and Lymphatic Vessel Density

Immunostaining for Ang-2 and VEGFR-3 was assessed in terms of staining intensity and the percentage of positive cells on light microscopy as described previously [7]. The scores for staining intensity were as follows: 0, no detectable staining; 1, faint staining; 2, moderate staining; and 3, strong staining. The scores for percentage of positive cells on light microscopy were as follows: 0, 0% to 10% of cells stained positive; 1, 11% to 30%; 2, 31% to 50%; and 3, 51% to 100%. Ang-2 or VEGFR-3 expression was classified as high if the total score was above the median of scoring of intensity or percentage of positive cells and low if the total score was below the median of scoring of intensity or percentage of positive cells.

Microvessel density (MVD) was determined by counting cells with positive staining for antibody to CD34 on light microscopy. Briefly, MVD was assessed by light microscopy in areas of tumor containing the highest numbers of small venules and capillaries per area (i.e., areas of the most intense neovascularization). The stained sections were screened at 100× magnification to identify the 3 regions of each section with the greatest numbers of distinct factor CD34 staining microvessels per area, termed vascular hot spots. The hotspots could appear anywhere within an invasive tumor but most frequently were seen at the margins of tumor. The vascular hot spots were believed to have higher malignant potential than other regions of tumor tissue [8]. Each endothelial cell or endothelial cell cluster with positive factor CD34 antigen was considered to be a countable, single microvessel (Fig. 1). The microvessels in each of these areas were then counted at 400× magnification (40× objective lens and 10× ocular lens, 0.189 mm2 per field). The average number of microvessels in these 3 regions was defined as a section MVD. Three discontinuous sections were obtained from each sample, of which, the highest section MVD was used to further analyze. A similar approach was used for the determination of lymphatic vessel density (LVD). The greatest numbers of distinct factor D2-40 staining lymphatic vessels per area were termed lymphatic hot spots.

### Statistical Analysis

Comparisons between the two groups (Ang-2 or VEGFR-3 expression) were performed using an independent two sample t test for continuous variables and Chi-square test for categorical variables. In addition, differences in MVD and LVD among the Ang-2 (high), Ang-2 (low), VEGF (high), and VEGF (low) groups were compared using one-way ANOVA. Continuous variables were represented as mean ± standard deviation (SD), and categorical data were represented by number (n) and percentage (%).

The primary endpoint in this study is overall death. Overall survival (OS) was defined as the time from first appointment to death from any cause or date of last follow-up. Participants who were alive at the end of the study period or lost to follow-up were considered censored. Medical record review for follow-up status of all patients was performed under direct supervision of staff head and neck surgeon. Primary tumor subsite, clinical stage, treatment, and vital status were reviewed from medical records as assessed between the initial and final patient contact recorded. The survival rates were calculated using the Kaplan–Meier method, and the differences between the survival curves were examined by the log-rank test. Univariate cox proportional hazards regressions were applied to estimate the individual hazard ratio (HR) for the overall survival. The significant variables in the univariate analyses (*P*<0.05) were then put into the multivariate analysis. The HR with 95% confidence interval was measured to estimate the hazard risk of individual factors. SPSS software, version 16.0 (SPSS, Inc. Chicago, IL, USA), was used for the statistical analyses. All P values are 2-sided, and statistical significance was defined as *P*<0.05.

## Results

### Patient Characteristics

The clinicopathological characteristics of the 112 patients with OSCC from whom tumor tissues were obtained are summarized in Table 1. Overall, the distribution of both expression of Ang-2 and VEGFR-3 had no significant differences in age, gender, lesion site, tumor differentiation, TNM stage, or smoking or alcohol use (all *P*>0.05) except Lymph node metastasis for VEGFR-3 (*P* = 0.000). Furthermore, we did not find a significant association of survival with most of these clinicopathological characteristics (log-rank, P>0.05); however, patients with poor/moderate differentiation and Lymph node metastasis had a significantly worse survival than those with high differentiation and without Lymph node metastasis (*P* = 0.016 for differentiation and *P* = 0.008 for Lymph node metastasis), respectively.

**Table 1:** Association of clinicopathologic characteristics with Ang-2 or VEGFR-3 expression and survival in 112 OSCC patients.

Clinicopathologic characteristic	n	Ang-2 expression		VEGFR-3 expression		Log-rank test
		n (%)	*P^1^*	n (%)	*P^1^*	P*
Age, years			0.252		0.127	0.287
≥56	48	27 (56.3)		26 (54.2)		
<56	64	29 (45.3)		28 (43.8)		
Gender			0.526		0.291	0.372
Female	31	17 (54.8)		13 (41.9)		
Male	81	39 (48.1)		43 (53.1)		
Lesion site			1.000		0.484	0.295
Other	76	38 (50.0)		35 (46.1)		
Tongue	36	18 (50.0)		16 (44.4)		
Smoking			0.477		0.751	0.298
Never	67	35 (52.2)		33 (49.3)		
Ever	42	19 (45.2)		22 (52.4)		
Missing	3					
Alcohol			0.366		1.000	0.713
Never	56	34 (60.7)		28 (50.0)		
Ever	50	26 (52.0)		25 (50.0)		
Missing	6					
Differentiation			0.563		0.847	0.016
High	67	32 (47.8)		33 (49.3)		
Poor/moderate	45	24 (53.3)		23 (51.1)		
TNM stage			0.186		0.850	0.135
I or II	57	32 (56.1)		28 (49.1)		
III or IV	55	24 (43.6)		28 (50.9)		
Lymph node metastasis			0.088		0.000	0.008
Absent	61	26 (42.6)		21 (34.4)		
Present	51	30 (58.8)		35 (68.6)		

*P^1^*: chi-square test and *P: log-rank test.

### Expression of Ang-2 and VEGFR-3 in OSCC and Normal Control Tissues

**Figure 2:**
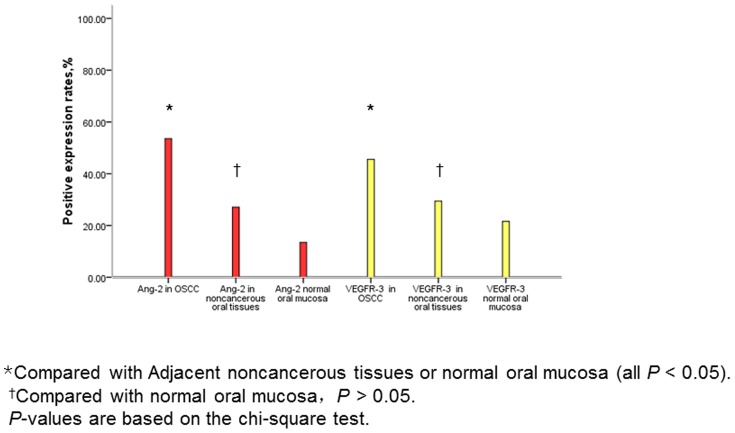
Rates of expression of Ang-2 and VEGFR-3 in OSCC and normal control tissues.

In general, in tumor cells, Ang-2 immunostaining in the cytoplasm was intense, while VEGFR-3 immunostaining in the cytoplasm was faint or moderate as shown in **Figure 1A**. Rates of Ang-2 and VEGFR-3 expression in OSCC samples and normal control tissues are shown in **Figure 2**. Ang-2 and VEGFR-3 expression were significantly more common in the 112 OSCC specimens (Ang-2: 60 specimens, 53.6%; VEGFR-3: 51 specimens, 45.5%) than in the 85 OSCC-adjacent noncancerous oral tissue specimens (Ang-2: 23, 27.0%; VEGFR-3: 25, 29.4%) or the 37 normal oral mucosa specimens (Ang-2: 5, 13.5%; VEGFR-3: 8, 21.6%) (all *P*<0.05). We found no significant difference in rates of Ang-2 and VEGFR-3 expression between OSCC-adjacent noncancerous tissues and normal oral mucosa samples (both *P*>0.05, **Figure 2**). However, we found that high expression of VEGFR-3 was significantly higher in OSCC patients with the presence of lymph node metastasis than those without lymph node metastasis (P = 0.000).

### Ang-2 and VEGFR-3 Expression and MVD and LVD in OSCC

Double staining with antibodies against CD34 and D2-40 revealed that CD34 was expressed in the erythrocyte-filled blood vessels whereas D2-40 was expressed exclusively in the lymphatic vessels in OSCC specimens (**Figure 1B**). Endothelial cells lining spaces devoid of blood cells (indicative of lymphatic spaces) exhibited strong D2-40 staining, whereas obvious blood vessels exhibited no D2-40 staining. Myoepithelial cells were also immunoreactive to D2-40, but myoepithelium could successfully be differentiated from lymphatic epithelium on the basis of location, organization, and size.

**Figure 3:**
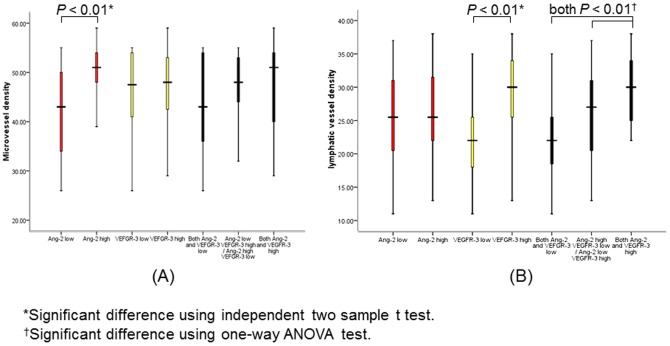
Correlation between Ang-2 and VEGFR-3 status with MVD (A) and LVD (B).

MVD was significantly greater in Ang-2-positive OSCC than in Ang-2-negative OSCC (mean [SD], 49.9 [6.0] vs 42.7 [9.0]; *P*<0.01), and no significant correlation was found between combined Ang-2 and VEGFR-3 expression and MVD (**Figure 3A**). LVD was significantly greater in VEGFR-3-positive OSCC than in VEGFR-3-negative OSCC (mean [SD], 29.7 [5.3] vs 22.2 [6.0]; *P*<0.01; **Figure 3B**). The mean LVD was higher for OSCC with high expression of both Ang-2 and VEGFR-3 (mean [SD], 29.6 [5.0]) than for OSCC with low expression of both Ang-2 and VEGF (mean [SD], 22.4 [6.3]; *P*<0.01) and OSCC with low Ang-2 expression and high VEGFR-3 expression or vice versa (mean [SD], 25.9 [6.8]; *P*<0.01). These results suggested that Ang-2 collaborates with VEGFR-3 in tumor lymphangiogenesis. In addition, no significant correlations were found between Ang-2 staining and LVD or between VEGFR-3 staining and MVD. Therefore, these results indicated that expression of Ang-2 in OSCC was closely related to angiogenesis whereas expression of VEGFR-3 in OSCC was closely related to lymphangiogenesis.

### Association of Ang-2 and VEGFR-3 Expression with survival in OSCC

**Figure 4:**
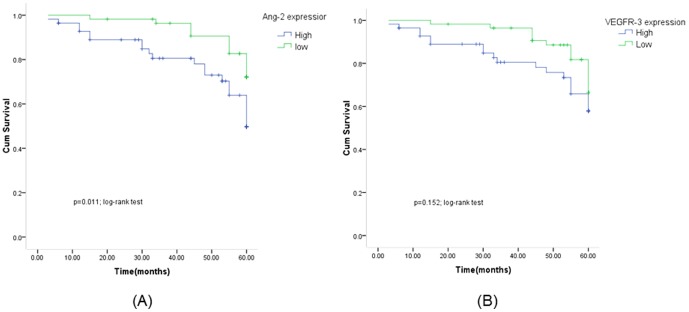
Kaplan-Meier survival for OSCC patients by Ang-2 (A) and VEGFR-3 (B) status.

**Figure 5:**
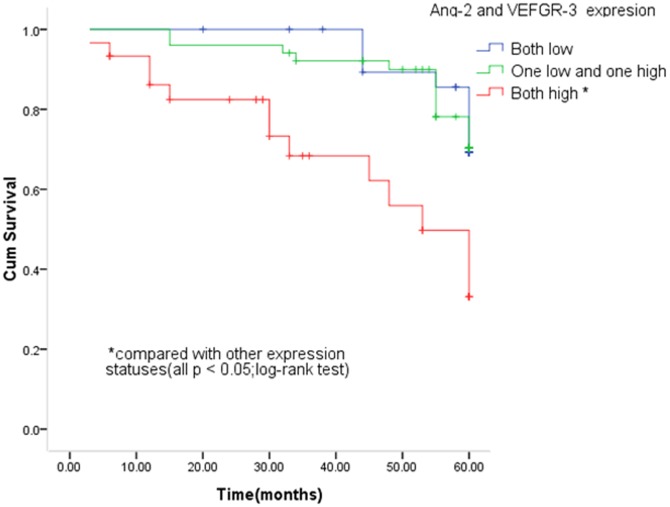
Kaplan-Meier survival for OSCC patients by combined Ang-2 and VEGFR-3 status.

**Figure 4** shows the univariate Kaplan-Meier analyses of survival with respect to the death from all causes. At a median follow-up time of 58 months (range, 3–60 months), 31 deaths from any causes occurred. The patients with high Ang-2 expression had a significantly worse overall survival than patients with low Ang-2 expression (*P* = 0.011, **Figure 4A**), while such a significant difference in survival was not found for VEGFR-3 expression (*P* = 0.152, **Figure 4B**). However, patients with high expression of both Ang-2 and VEGFR-3 had a significantly worse survival than patients with low expression of either of genes or any other co-expression status of both genes (*P*<0.05, **Figure 5**). Furthermore, the results of multivariable Cox proportional hazards regression analysis regarding the association between expression of both genes and risk of overall death are shown in **Table 2**. Estimates of association were adjusted for potential confounders including age, gender, tumor differentiation, TNM stage, postoperative treatment, smoking and alcohol use, and Lymph node metastasis. Compared with patients having low expression of either of genes, the patients with high expression had significantly increased risk of overall death (HR, 2.7; 95% CI, 1.6–6.2 for Ang-2 and 1.8, 0.7–4.7 for VEGFR-3). The increased risk for overall death was even higher for patients with high expression of both genes (HR, 5.0; 95% CI, 1.3–15.4) compared with any other co-expression status of both genes (**Table 2**).

**Table 2:** Univariate and multivariable analysis on association of Ang-2 and VEGFR-3 expression with survival of OSCC patients.

Ang-2/VEGFR-3 expression	n	Univariate analysis		Multivariate analysis*	
		HR (95% CI)	*P*	HR (95% CI)	*P*
Ang-2 expression					
Low	56	1		1	
High	56	2.5 (1.2–5.2)	0.013	2.7 (1.1–6.2)	0.025
VEGFR-3 expression					
Low	56	1		1	
High	56	2.0 (0.9–4.1)	0.059	1.8 (0.7–4.7)	0.204
Ang-2 and VEGFR-3 expression					
Both low	31	1		1	
One low and one high	51	1.3 (0.5–3.4)	0.638	1.3 (0.4–4.1)	0.662
Both high	30	5.1 (1.9–13.5)	0.001	5.0 (1.3–15.4)	0.016

*Adjusted for age, gender, tumor differentiation, TNM stage, postoperative treatment, smoking and alcohol use, and Lymph node metastasis.

## Discussion

The current study showed that high expression of Ang-2 individually, or in combination with VEGFR-3, was significantly associated with survival or increased risk for overall death of OSCC patients. The study is the first to demonstrate that expression of Ang-2 and VEGFR-3 could serve as prognostic markers for survival of OSCC patients. Such a biomarker might potentially be used to optimize OSCC patient stratification for personalized treatment and improved survival. However, how the overexpression of these two genes contributes to cancer progression/prognosis is not fully clear. Several mechanisms have been postulated. Lymphangiogenesis in tumor probably is mainly responsible for such prognosis since both genes play important roles in regulation of lymphangiogenesis [9].

Ang-2 is considered to be required for postnatal angiogenesis and lymphatic patterning [2]. However, the role of Ang-2 in lymphangiogenesis of OSCC is still unknown. In the present study, we found that expression levels of Ang-2 and VEGFR-3 were higher in OSCC than in OSCC-adjacent noncancerous oral tissues and normal oral mucosa. There was no significant difference in Ang-2 or VEGFR-3 expression between OSCC-adjacent noncancerous oral tissues and normal oral mucosa. In OSCC, high expression of VEGFR-3 was significantly correlated with the presence of lymph node metastasis. MVD was significantly higher in OSCC with high Ang-2 expression than in OSCC with low Ang-2 expression, and LVD was significantly higher in OSCC with high VEGFR-3 expression than in OSCC with low VEGFR-3 expression. Our results indicated that OSCC with up-regulated Ang-2 and VEGFR-3 expression induced tumor angiogenesis and lymphangiogenesis, which promoted OSCC. Further, we examined the relationships between Ang-2 and VEGFR-3 expression and lymphangiogenesis and angiogenesis in OSCC. We found that OSCC with high expression of both Ang-2 and VEGFR-3 had significantly higher LVD than OSCC with any other combination of Ang-2 and VEGFR-3 expression, whereas high expression of Ang-2 by itself was not associated with LVD or lymph node metastasis. Also, we found no significant correlation between high expression of both Ang-2 and VEGFR-3 and MVD. These results suggest that Ang-2 in collaboration with VEGFR-3, but not Ang-2 alone, promotes tumor lymphangiogenesis in OSCC and Ang-2 might promote VEGFR-3-related tumor lymphangiogenesis and lymph node metastasis in patients with OSCC.

To date, few studies on effect of tumor lymphangiogenesis and lymphatic capillary proliferation on cancer prognosis have been reported due to the lack of specific lymphatic endothelial markers and lymphatic-specific growth factors. D2-40 was reported to be selective for lymphatic endothelium in 2005 [10] and is known to selectively stain lymphatic endothelial cells, identifying lymphatic channels within tumor and in the peritumoral stroma [11]. Moreover, D2-40 has identified lymphatic invasion in many malignant tumors [10], [12].Our results showed that there was no immunologic cross-reactivity between antibodies against D2-40 and CD34. Double staining revealed exclusive expression of D2-40 in the lymphatic vessels, whereas CD34 was expressed in the erythrocyte-filled blood vessels. LVD, as a quantitative parameter of tumor lymphangiogenesis, is an important prognostic factor associated with lymph node metastasis, distant metastasis, and poor prognosis [13].

Increased LVD is associated with many newly identified lymphatic growth factors, including VEGFR-3. VEGFR-3 is considered a major regulator of lymphangiogenesis [4]. By blocking VEGFR-3 signaling, lymphangiogenic effect can be suppressed [14]. VEGFR-3 expression was significantly associated with LVD in invasive squamous cell cervical cancer and upregulation of VEGFR-3 early in the carcinogenic process may indicate a more aggressive phenotype with a higher propensity for early lymphatic dissemination [15]. Inhibition of lymphangiogenesis by targeting VEGFR-3 phosphorylation is considered to be a therapeutic strategy for inhibiting lymph node metastasis of diffuse-type gastric cancer [16].Previous studies by our group and others have suggested that Ang-2 might, in some cases, collaborate with VEGF to promote tumor angiogenesis [3], [17]. However, the relationship between VEGFR-3 and Ang-2 in tumor lymphangiogenesis and lymph node metastasis remains further investigation.

Ang-2 signaling may play critical roles in the lymphatic vessel system. It was previously reported that Ang-2 is required to form functional lymphatics [2]. However, unlike other lymphatic regulators, such as VEGFR-3 or the transcription factor Prox-1 pathway [18], [19], Ang-2 appears not to be required for initiation of lymphatic vascular development. Rather, Ang-2 seems to play a key role in remodeling and maturation of the lymphatics. This concept is supported by the phenotype of Ang-2 knockout mice, which exhibit hypoplasia of the lymphatic capillaries in the skin and small intestine and have collecting lymphatic vessels not properly invested by smooth muscle cells [2]. To the best of our knowledge, the current study is the first to examine whether Ang-2 expression and VEGFR-3 expression predict overall survival in patients with OSCC with long-term follow-up. Both univariate and multivariable analyses showed that patients with high expression of Ang-2 or both Ang-2 and VEGFR-3 was significantly associated with survival or risk of overall death compared with those with low expression of Ang-2 or other combinations of Ang-2 and VEGFR-3 expression, indicating that high expression of both Ang-2 and VEGFR-3 in tumor cells predicts poor clinical outcome in patients with OSCC. A recent study [20] has shown that systemic overexpression of Ang-2 promoted metastatic dissemination whereas specific Ang-2 blockade attenuated tumor lymphangiogenesis and reduced tumor cell dissemination into the regional lymph nodes. Metastasis to regional lymph nodes constitutes the main route toward progression and dissemination of OSCC; at the same time it is the most significant adverse prognostic indicator for this disease. In recent years, significant effort has been expended in an effort to clarify the molecular mechanisms underlying lymph node metastasis of oral cancer. Our studies demonstrated that high expression of Ang-2 cooperated with VEGFR-3 in OSCC and was significantly associated with LVD and poor prognosis. Ang-2 may play a crucial role in the VEGFR-3-related tumor lymphangiogenesis and progression of OSCC. The precise nature of the collaboration between Ang-2 and VEGFR-3 remains a mystery, but one which must be solved to allow for the development of drugs targeting these factors to prevent lymphangiogenesis. Further research is necessary to uncover the molecular pathways of lymphangiogenesis and develop novel therapeutic options for OSCC. In conclusion, our results demonstrated that high expression of Ang-2 alone or in combination with VEGFR-3 in OSCC was significantly associated with prognosis of OSCC patients. Further larger studies are required for validation of our findings and an exploration of the molecular mechanisms underlying the observed associations.
